# Mitochondrial Morphology and TOMM20/TIMM44 Abundance During Prolonged HEK293 Cell Culture Under Different Glucose and Medium-Replacement Conditions

**DOI:** 10.3390/cells15212031

**Published:** 2026-11-07

**Authors:** Kiyoharu Kawakami, Mirai Koike, Nozomi Ito, Rei Watanabe, Takahiro Tsukimura, Kazuhiko Nakadate

**Affiliations:** Department of Functional Morphology, Meiji Pharmaceutical University, 2-522-1 Noshio, Kiyose, Tokyo 204-8588, Japan; k-kawakami@my-pharm.ac.jp (K.K.); y211105@std.my-pharm.ac.jp (M.K.); m256206@std.my-pharm.ac.jp (N.I.); m266234@std.my-pharm.ac.jp (R.W.); takahiro@my-pharm.ac.jp (T.T.)

**Keywords:** glucose availability, medium replacement, mitochondrial remodeling, mitochondrial morphology, TOMM20, TIMM44, mitochondrial protein import, extracellular environment, HEK293 cells, transmission electron microscopy

## Abstract

**Highlights:**

**What are the main findings?**

- Prolonged high-glucose culture increased TOMM20 and TIMM44 abundance and was associated with a larger mitochondrial cross-sectional area in HEK293 cells, without a significant change in mitochondrial density.
- With daily medium replacement, differences in TOMM20 and TIMM44 abundance were no longer detected, whereas the difference in mitochondrial cross-sectional area between standard- and high-glucose conditions was reduced but remained detectable.

**What are the implications of the main findings?**

- The medium-replacement schedule can influence the mitochondrial changes attributed to glucose conditions during prolonged cell culture.
- Changes in the extracellular culture environment should be considered when interpreting mitochondrial responses to glucose levels in vitro.

**Abstract:**

Glucose availability is essential for cellular metabolism and maintaining mitochondrial homeostasis. However, the effects of sustained changes in glucose availability on mitochondrial protein abundance and morphology remain unclear. In this study, we examined the mitochondrial responses to different glucose concentrations and medium replacement conditions. HEK293 cells were cultured for 4 days in standard or high-glucose medium without medium replacement. TOMM20 and TIMM44 abundance was evaluated using immunofluorescence and Western blotting, and mitochondrial morphology was assessed using transmission electron microscopy. The mitochondrial cross-sectional area and density were quantified. Glucose concentrations and medium pH were monitored throughout the culture period. In a separate experiment, the culture medium was replaced every 24 h. Without medium replacement, high-glucose culture was associated with greater TOMM20 and TIMM44 abundance and larger mitochondria than standard-glucose culture, whereas mitochondrial density did not differ significantly. With daily medium replacement, differences in TOMM20 and TIMM44 abundance were no longer detected, and mitochondrial size did not differ significantly between the glucose conditions. Moreover, mitochondrial density did not differ significantly between the glucose conditions. These findings indicate that mitochondrial morphology and TOMM20/TIMM44 abundance during prolonged culture are associated with both the initial glucose concentration and subsequent changes in the extracellular culture environment.

## 1. Introduction

Glucose availability is a key determinant of cellular metabolism, growth, and homeostasis. Mammalian cells continuously adjust their metabolism in response to changes in extracellular nutrient availability, and mitochondria coordinate cellular energy metabolism with biosynthetic and stress-responsive processes [1,2]. Mitochondrial morphology and molecular composition undergo dynamic remodeling in response to changes in the cellular environment [2,3,4]. Although high-glucose conditions are commonly used to study metabolic stress and glucose-related cellular responses, the effects of prolonged changes in glucose availability on mitochondrial morphology and protein composition remain unclear.

Mitochondria are dynamic organelles that undergo continuous structural remodeling in response to cellular metabolic demands. Mitochondrial fusion, fission, biogenesis, and degradation maintain mitochondrial homeostasis and enable cellular adaptation to environmental changes [2,4,5]. Morphological changes in mitochondria, including enlargement and fragmentation, occur in various physiological and pathological conditions. These alterations may be associated with changes in cellular metabolism and mitochondrial homeostasis [2,6,7]. Ultrastructural analysis can be used to characterize mitochondrial remodeling in response to metabolic stress [8,9]. However, morphological changes do not necessarily indicate altered mitochondrial functions [6,10,11]. Therefore, combining ultrastructural analysis with measurements of mitochondrial protein abundance can provide a more complete characterization of mitochondrial remodeling.

Mitochondrial homeostasis relies on the continuous import of proteins synthesized in the cytosol. More than 99% of mitochondrial proteins are encoded by nuclear DNA and synthesized as precursor proteins in the cytoplasm [12,13,14]. Coordinated translocation systems in the outer and inner mitochondrial membranes transport these proteins into the mitochondria. TOMM20 is the principal receptor in the translocase of the outer mitochondrial membrane (TOM) complex and recognizes precursor proteins targeted to the mitochondria [13]. TIMM44 is associated with the translocase of the inner mitochondrial membrane (TIM23) complex and facilitates the translocation of precursor proteins into the mitochondrial matrix [15]. Changes in TOMM20 and TIMM44 abundance may reflect alterations in mitochondrial protein homeostasis and remodeling. An increase in the abundance of these proteins does not necessarily indicate a greater mitochondrial quantity because it may instead reflect changes in the protein composition or state of individual mitochondria [16,17,18].

Nutrient availability influences mitochondrial metabolism, morphology, and homeostasis [2,5]. Accordingly, prolonged cell culture experiments require careful interpretation when assessing the effects of glucose concentration on mitochondrial remodeling. In culture systems without medium replacement, cells consume glucose and release metabolic byproducts into the medium [19,20]. Consequently, the extracellular environment changes progressively during culture, including changes in glucose availability and medium pH [19,21]. The initial glucose concentration may therefore not represent the extracellular environment experienced by cells throughout a prolonged culture period [19,20,21,22]. These temporal changes may affect mitochondrial morphology and protein composition. They also complicate efforts to distinguish responses to the initial glucose concentration from those associated with subsequent changes in the culture environment.

This issue is especially pertinent when comparing standard- and high-glucose conditions during prolonged culture. Cells in high-glucose medium are initially exposed to a greater glucose concentration than those in standard-glucose medium. However, cells consume glucose under both conditions. The resulting reduction in glucose availability may alter the cellular metabolic environment over time [23]. Periodic medium replacement enables comparison of mitochondrial responses when glucose is replenished and accumulated extracellular components are removed. This comparison may help clarify how changing culture conditions are associated with mitochondrial remodeling during prolonged culture.

HEK293 cells, which are derived from human embryonic kidney tissue, are widely used immortalized cells characterized by robust growth and active metabolism [22]. Their consistent growth under defined culture conditions makes them suitable for examining cellular and mitochondrial responses to changes in nutrient availability [24]. Although HEK293 cells do not represent normal differentiated kidney cells, they provide a model for examining cellular adaptation to different glucose environments. This study employed HEK293 cells to examine the morphological and molecular responses of mitochondria during extended culture under diverse glucose conditions.

In this study, we aimed to examine mitochondrial morphology and protein abundance in HEK293 cells during prolonged culture under standard glucose (SG; 5.5 mM) and high-glucose (HG; 25 mM) conditions. Cellular morphology and mitochondrial staining were initially assessed using Giemsa and Janus Green staining, respectively. TOMM20 and TIMM44 abundance was then evaluated by immunofluorescence staining and Western blotting. Mitochondrial ultrastructure and cross-sectional area were assessed using transmission electron microscopy. Glucose concentrations and medium pH were monitored throughout the culture to characterize changes in the extracellular environment. To compare mitochondrial responses when the extracellular environment was periodically renewed, a separate experiment was conducted in which the culture medium was replaced every 24 h with fresh medium containing the corresponding glucose concentration. By integrating cellular, molecular, and ultrastructural analyses with measurements of the extracellular environment, this study aimed to determine how glucose and medium-replacement conditions relate to mitochondrial morphology and TOMM20/TIMM44 abundance during prolonged culture.

## 2. Materials and Methods

### 2.1. Cell Culture and Glucose Conditions

Human embryonic kidney 293 cells (HEK293; ATCC CRL-1573) were cultured in Dulbecco’s modified Eagle’s medium (DMEM) supplemented with 10% heat-inactivated fetal bovine serum at 37 °C in a humidified atmosphere containing 5% CO_2_. High-glucose DMEM (FUJIFILM Wako Pure Chemical Corporation, Osaka, Japan; Cat. No. 043-30085), containing 4.5 g/L glucose, and low-glucose DMEM (FUJIFILM Wako Pure Chemical Corporation; Cat. No. 041-29775), containing 1.0 g/L glucose, were used for the high-glucose (HG) and standard-glucose (SG) conditions, respectively. According to the manufacturer’s published composition tables, the two DMEM formulations have the same composition except for their glucose concentrations.

In this study, cells were cultured under high-glucose (HG; 4.5 g/L D-glucose) or standard-/low-glucose (SG; 1.0 g/L D-glucose) conditions. Cells were seeded in 6 cm culture dishes at a density of 1.0 × 10^5^ cells per dish in 4 mL of culture medium and cultured for 4 days without medium replacement. This design allowed the glucose and other components of the extracellular environment to change progressively during the culture period.

### 2.2. Measurement of Extracellular Glucose Concentration and pH

Residual glucose concentrations in the culture medium were measured throughout the culture period. In cultures maintained for 4 days without medium replacement, glucose concentrations were measured daily. For cultures with daily medium replacement, glucose concentrations were measured immediately before and after each medium exchange during the four-day experimental period. A 1.5 μL aliquot of culture medium was collected and analyzed using Glucose Test Strips with the GlucCell^®^ system (CESCO Bioengineering Co., Ltd., Taichung, Taiwan). Measurements were performed using three independent culture dishes for each condition. Glucose concentration and pH were measured repeatedly from the same culture dishes at successive time points. Separate culture dishes were not used for each time point.

The pH of the culture medium was measured throughout the culture period using a pH meter (LAQUAtwin pH-33, HORIBA, Kyoto, Japan). Before use, the pH meter was calibrated using the manufacturer’s standard calibration solutions. Culture dishes were briefly removed from the incubator, and the pH of the culture medium was measured in the laboratory. For cultures maintained for 4 days without medium replacement, pH was measured daily. For cultures with daily medium replacement, the pH was measured immediately before and after each medium exchange during the four-day experimental period. Measurements were performed using three independent culture dishes for each condition in each experiment.

### 2.3. Cellular and Mitochondrial Staining

Giemsa and Janus Green B staining were performed to assess cellular morphology under different glucose conditions. Cells were fixed with 4% paraformaldehyde (PFA) for 15 min and then stained with Giemsa solution (MUTO PURE CHEMICALS CO., LTD., Tokyo, Japan). Giemsa staining was performed to qualitatively examine general cellular morphology under the different glucose culture conditions. The stained cells were examined for overall appearance and morphological changes. No quantitative analysis of cell number or cell area was performed using the Giemsa-stained images. For mitochondrial visualization, cells were stained with Janus Green B (FUJIFILM Wako Pure Chemical Corporation) according to the manufacturer’s instructions and examined using a light microscope (BZ-X710; Keyence, Osaka, Japan).

### 2.4. Immunofluorescence Staining

Cells were cultured for 4 days on coverslips in 6 cm dishes and then fixed with 4% paraformaldehyde (PFA) for 15 min. After permeabilization with phosphate-buffered saline (PBS) containing 0.1% Triton X-100 for 10 min, the cells were blocked with 5% bovine serum albumin (BSA) for 1 h. The cells were incubated overnight at 4 °C with primary antibodies against TOMM20 (1:500; ab186734; Abcam, Cambridge, UK) and TIMM44 (1:500; PA5-106383; Thermo Fisher Scientific, Waltham, MA, USA). After washing, the cells were incubated with Alexa Fluor 488-conjugated secondary antibodies (1:2000; Proteintech Group, Inc., Rosemont, IL, USA) for 1 h at room temperature, in the dark. Nuclei were counterstained with 4′,6-diamidino-2-phenylindole (DAPI; Merck KGaA, Darmstadt, Germany). Fluorescence images were acquired using a fluorescence microscope (BZ-X710; Keyence, Osaka, Japan). Immunofluorescence staining for TOMM20 and TIMM44 was performed in three independent cell culture experiments under the same experimental conditions as those used for Western blot analysis. The images were used for qualitative assessment of staining patterns and cellular localization. Fluorescence intensity was not quantitatively analyzed.

### 2.5. Western Blot Analysis

Cells were lysed using RIPA buffer containing protease inhibitors (EzRIPA Lysis Kit; ATTO, Tokyo, Japan). Protein concentrations were measured using a BCA Protein Assay Kit (Thermo Fisher Scientific). Equal amounts of protein (20 μg per sample) were separated by SDS-PAGE on 5–20% gradient gels (e-PAGEL; ATTO) and transferred to PVDF membranes using the QBlot Kit M (ATTO). After transfer, the membranes were stained with EzStain AQua MEM (WSE-7160; ATTO) to visualize total protein. The membranes were destained according to the manufacturer’s instructions and then blocked and incubated with antibodies. The membranes were blocked with EzBlock Chemi (ATTO) for 30 min at room temperature and incubated overnight at 4 °C with primary antibodies against TOMM20 (1:10,000; ab186734; Abcam) and TIMM44 (1:1000; PA5-106383; Thermo Fisher Scientific). After incubation with horseradish peroxidase (HRP)-conjugated secondary antibodies (1:20,000; Proteintech Group, Inc., Rosemont, IL, USA), signals were detected using ImmunoStar LD chemiluminescent reagent (FUJIFILM Wako Pure Chemical Corporation).

The total protein signal in each lane of the EzStain AQua MEM-stained membrane was used as a loading control. Target protein band intensities were normalized to the corresponding total protein signal in the same lane.

For TOMM20 and TIMM44, the bands at approximately 20 kDa and 44 kDa, respectively, corresponding to their expected molecular weights, were selected for densitometric quantification. The arrows in the figures indicate the bands used for quantification. Band intensities were quantified using ImageJ (version 1.54j). The intensity of each target protein band was normalized to the corresponding total-protein signal detected using EzStain AQua MEM.

### 2.6. Transmission Electron Microscopy Analysis

Transmission electron microscopy (TEM) was used to assess the mitochondrial morphology. Cells were fixed in 2% paraformaldehyde and 2% glutaraldehyde for 2 h and post-fixed in 1% osmium tetroxide. The samples were dehydrated using a graded ethanol series and embedded in epoxy resin. Ultrathin sections (70 nm thick) were prepared and stained with uranyl acetate and lead citrate, as described previously. Images were acquired using a transmission electron microscope (HT-7800; Hitachi, Tokyo, Japan) at an accelerating voltage of 80 kV.

Mitochondrial cross-sectional areas were measured in the TEM images. For each experimental group, three independent dishes were analyzed, with at least 50 cells randomly selected from each dish. Approximately 100 mitochondria were randomly selected from each of the three independent culture dishes, resulting in approximately 300 mitochondria analyzed per group. The mean mitochondrial cross-sectional area was calculated for each dish, and the dish-level means were used for between-group statistical comparisons. Mitochondrial areas were measured using ImageJ software.

Mitochondrial density was quantified from TEM images and expressed as the number of mitochondria per 100 μm^2^ of cellular area. For each experimental group, three independent culture dishes were analyzed. At least 30 cells were randomly selected from each dish, and a total of 100 cells were analyzed per condition. TEM images were acquired from randomly selected areas. Images for analysis were randomly selected from the acquired images, and all mitochondria identifiable within the selected images were included in the analysis. Only one image per cell was used to avoid duplicate sampling of the same cell. For each analyzed cell, the number of mitochondria and corresponding cellular area were determined, and mitochondrial density was calculated as the number of mitochondria per 100 μm^2^ of cytoplasmic area. Cytoplasmic area was defined as the area of the cytoplasm visible in each TEM image, excluding the nucleus and extracellular regions. The mean mitochondrial density was then calculated for each independent culture dish, and dish-level means were used for between-group statistical comparisons. Mitochondrial area and cellular area were measured using ImageJ software.

### 2.7. Statistical Analysis

For morphometric analyses, the mean mitochondrial cross-sectional area and mean mitochondrial density calculated for each independent culture dish were treated as independent observations (n = 3 dishes per condition). For mitochondrial density analysis, individual cells were measured within each dish, and the mean value for each dish was used as the independent observation for statistical analysis.

For Western blot analysis, four independent samples were assessed for each experimental group (n = 4). Protein band intensities were quantified using densitometry and normalized to the corresponding total-protein signal. Differences between two groups were assessed using an unpaired two-tailed Student’s *t*-test, where appropriate. For comparisons among the four culture conditions in Figure 8A, one-way analysis of variance (ANOVA) followed by Tukey’s multiple-comparison test was performed. In addition, two-way ANOVA was performed to evaluate the main effects of glucose concentration and medium-replacement condition, as well as their interaction, on mitochondrial cross-sectional area. Statistical analyses were performed using StatView software (version 5.0, SAS Institute Inc., Cary, NC, USA). Data are presented as the mean ± standard deviation (SD), and *p* < 0.05 was considered statistically significant.

## 3. Results

### 3.1. High-Glucose Conditions Induced Morphological Changes in HEK293 Cells

Cellular morphology was compared after HEK293 cells were cultured for 4 days under standard-glucose (SG; 1 g/L, 5.5 mM) or high-glucose (HG; 4.5 g/L, 25 mM) conditions.

**Figure 1:**
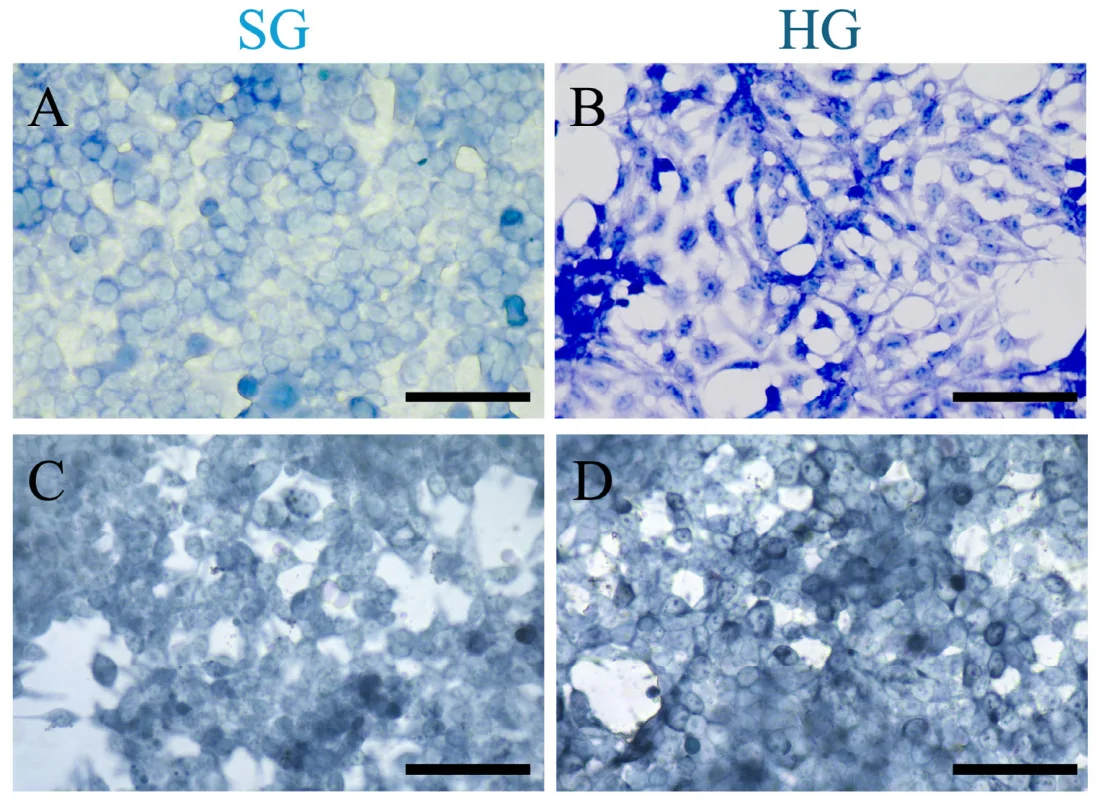
Cellular morphology and mitochondrial staining of HEK293 cells cultured under standard-glucose (SG; 1.0 g/L, 5.5 mM) or high-glucose (HG; 4.5 g/L, 25 mM) conditions for 4 days without medium replacement. (**A**,**B**) Representative images of Giemsa-stained cells cultured under SG and HG conditions. (**C**,**D**) Representative images of Janus Green B-stained cells cultured under SG and HG conditions. Scale bars, 50 μm.

Giemsa staining revealed differences in cellular morphology between the two groups. Compared with SG cells, HG cells showed more intense basophilic staining of the nuclei and nucleoli. Nucleolar staining appeared more intense under HG conditions, whereas cytoplasmic staining appeared less intense (Figure 1A,B). These morphological differences prompted further evaluation of the mitochondria under the two glucose conditions.

Janus Green B staining appeared more pronounced in HG cells than in SG cells (Figure 1C,D). However, the difference was not sufficiently distinct for reliable quantification. Mitochondrial alterations were examined further using TOMM20 immunofluorescence staining.

Prolonged culture under different glucose and medium-replacement conditions was associated with changes in HEK293 cell morphology. However, the vacuole-like structures observed using light microscopy could not be definitively identified, and their origin remains unclear. These morphological changes may reflect the combined effects of prolonged culture, altered nutrient availability, and changes in the extracellular environment.

### 3.2. High-Glucose Exposure Increased TOMM20 and TIMM44 Protein Abundance

**Figure 2:**
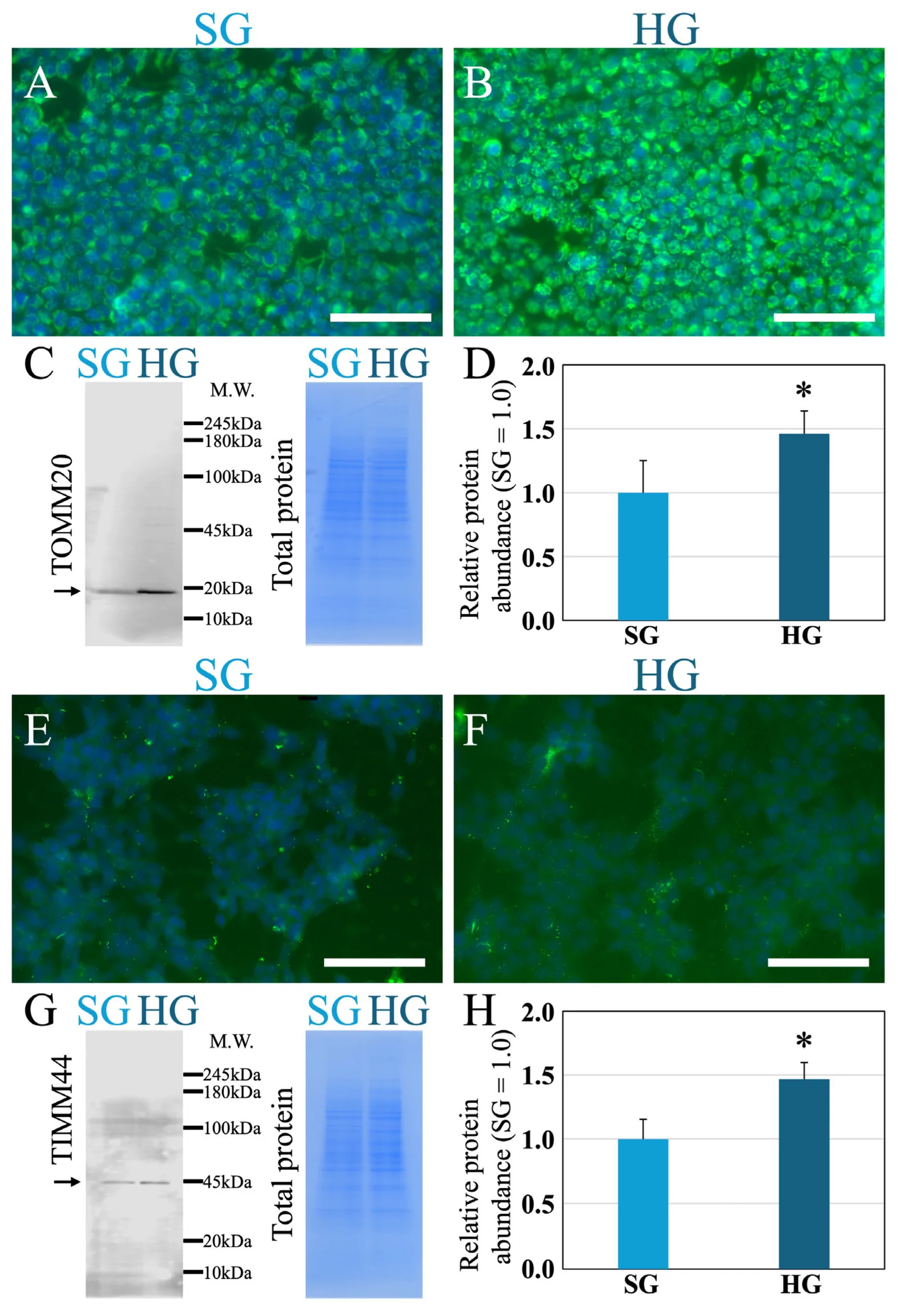
TOMM20 and TIMM44 protein abundance in HEK293 cells cultured under standard-glucose (SG) or high-glucose (HG) conditions for 4 days without medium replacement. (**A**,**B**) Representative TOMM20 immunofluorescence images. Nuclei were counterstained with DAPI. (**C**) TOMM20 Western blot and corresponding total protein staining with EzStain AQua MEM. The arrow indicates the TOMM20 band at approximately 20 kDa used for densitometric quantification. (**D**) TOMM20 abundance normalized to the total protein signal and expressed relative to the SG group, which was set to 1.0. (**E**,**F**) Representative TIMM44 immunofluorescence images. Nuclei were counterstained with DAPI. (**G**) TIMM44 Western blot and corresponding total protein staining with EzStain AQua MEM. The arrow indicates the TIMM44 band at approximately 44 kDa used for densitometric quantification. (**H**) TIMM44 abundance normalized to the total protein signal and expressed relative to the SG group, which was set to 1.0. EzStain AQua MEM total protein staining served as the loading control, and target protein band intensities were normalized to the total protein signal. Representative immunofluorescence images are shown for qualitative assessment; fluorescence intensity was not quantitatively analyzed. Data are presented as mean ± SD (n = 4 independent samples per group). M.W. indicates molecular weight in kilodaltons (kDa). * *p* < 0.05 versus the SG group. Scale bars, 50 μm.

Mitochondrial structures were initially visualized using Janus Green B staining. However, this staining did not clearly distinguish between SG and HG cells. Therefore, TOMM20 and TIMM44, components of the mitochondrial protein-translocation machinery, were examined using immunofluorescence staining (Figure 2).

TOMM20 fluorescence was more intense in HG cells than in SG cells (Figure 2A,B). TOMM20 abundance was evaluated using Western blotting with EzStain AQua MEM total-protein normalization (Figure 2C). Consistent with the immunofluorescence images, total protein-normalized TOMM20 abundance was significantly higher in HG cells than in SG cells (n = 4, *p* < 0.05; Figure 2D).

In contrast, TIMM44 fluorescence did not differ visibly between the SG and HG cells (Figure 2E,F). Nevertheless, Western blotting showed that TIMM44 abundance normalized to total protein was significantly higher in HG cells than in SG cells (n = 4, *p* < 0.05; Figure 2G,H). For TIMM44, densitometric analysis was performed using only the band corresponding to the expected molecular weight of the protein. The molecular weight is indicated in Figure 2.

Representative immunofluorescence images were used to illustrate the staining patterns and cellular localization of TOMM20 and TIMM44. Fluorescence intensity was not quantitatively analyzed. Differences in protein abundance were evaluated quantitatively by Western blotting. Collectively, prolonged culture under HG conditions was associated with increased TOMM20 and TIMM44 abundance in HEK293 cells. However, the increase in TIMM44 abundance was not evident in immunofluorescence images. These results indicate that prolonged HG culture is associated with altered abundance of mitochondrial protein translocation components.

### 3.3. High-Glucose Exposure Was Associated with Mitochondrial Enlargement on Transmission Electron Microscopy

**Figure 3:**
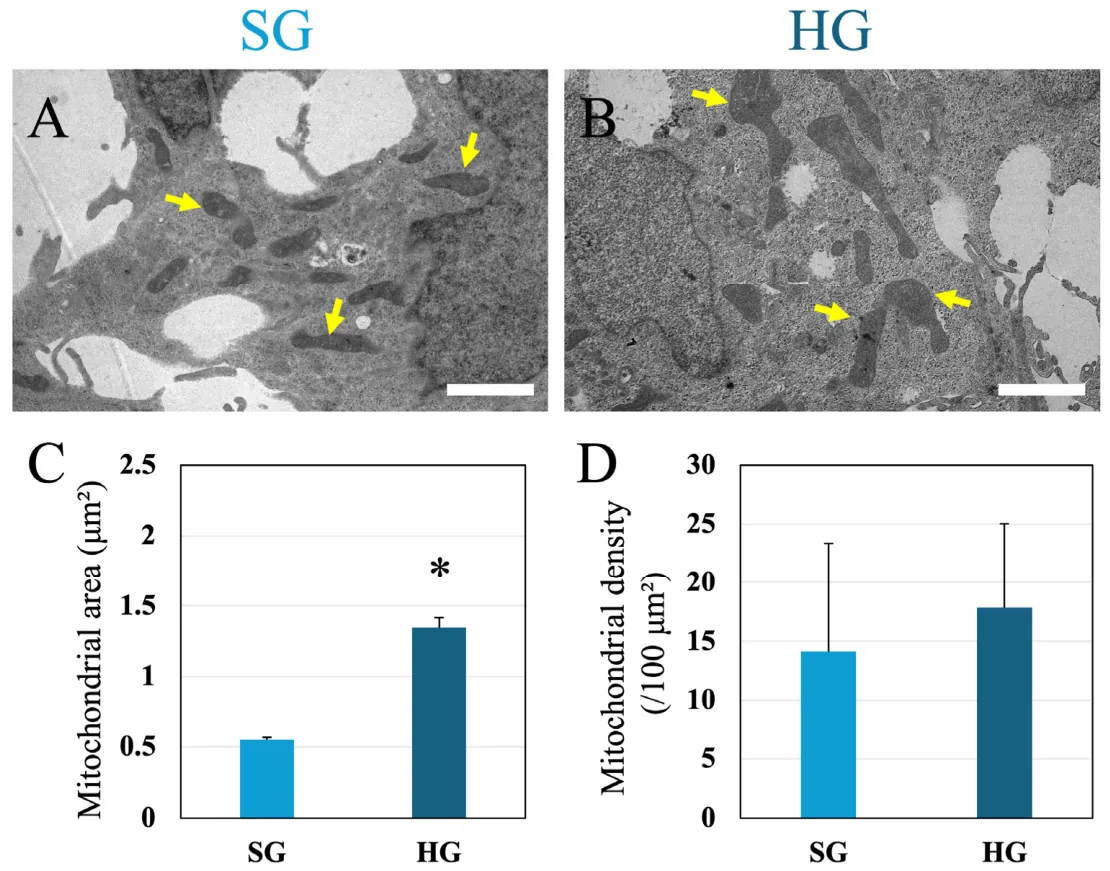
Mitochondrial morphology and density in HEK293 cells cultured under standard- and high-glucose conditions. (**A**,**B**) Representative transmission electron microscopy (TEM) images of HEK293 cells cultured under standard-glucose (SG; 1.0 g/L, 5.5 mM) or high-glucose (HG; 4.5 g/L, 25 mM) conditions for 4 days without medium replacement. Arrows indicate mitochondria. (**C**) Quantification of mitochondrial cross-sectional area. (**D**) Quantification of mitochondrial density, expressed as the number of mitochondria per 100 μm^2^ of cellular area. Data are presented as mean ± SD (n = 3 independent dishes per group). * *p* < 0.05 versus the SG group. Scale bars, 2 μm.

The mitochondrial ultrastructure was examined using transmission electron microscopy to determine whether the increased protein abundance was accompanied by morphological changes (Figure 3A,B). The cross-sectional areas of approximately 300 mitochondria per group were measured. The mean mitochondrial area was significantly greater in the HG group than in the SG group (Figure 3C; n = 3 independent dishes per group; *p* < 0.05). The mean mitochondrial area under HG conditions was more than twice that under SG conditions.

To determine whether the increase in mitochondrial size was accompanied by a change in mitochondrial density, the number of mitochondria per 100 μm^2^ of cellular area was quantified (Figure 3D). A total of 100 cells per condition were analyzed from three independent culture dishes, with at least 30 cells randomly selected from each dish. Mitochondrial density did not differ significantly between the SG and HG groups (n = 3 independent dishes per group; *p* > 0.05). Thus, prolonged HG culture was associated with mitochondrial enlargement without a detectable increase in mitochondrial density.

### 3.4. Changes in Glucose Concentration and pH During Prolonged Culture

Residual glucose concentration and medium pH were monitored over 4 days to characterize changes in the extracellular culture environment. Measurements were obtained under standard-glucose (SG; 1 g/L, 5.5 mM) and high-glucose (HG; 4.5 g/L, 25 mM) conditions without medium replacement.

**Figure 4:**
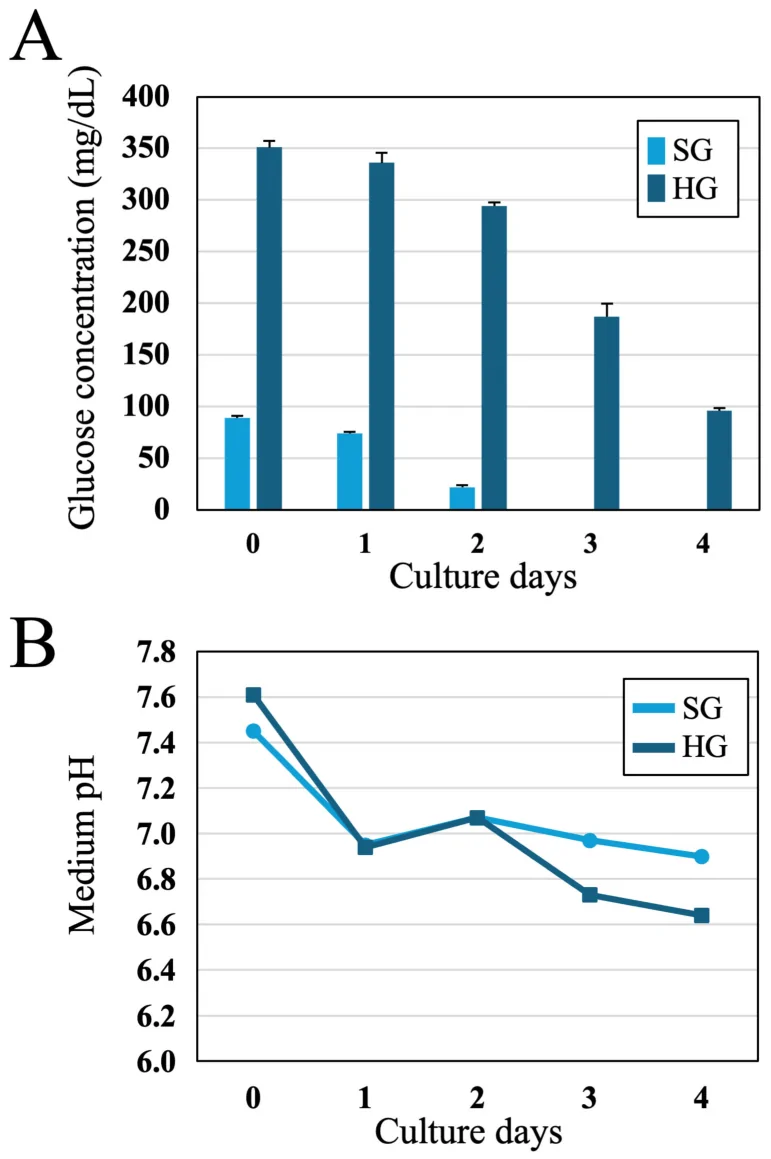
Changes in residual glucose concentration and medium pH during prolonged culture without medium replacement. HEK293 cells were cultured under standard-glucose (SG) or high-glucose (HG) conditions for 4 days without medium replacement. (**A**) Residual glucose concentration. (**B**) Medium pH. Data are presented as mean ± SD (n = 3).

Residual glucose concentrations progressively decreased under both SG and HG conditions (Figure 4A). The decline was more pronounced under SG conditions, with residual glucose levels approaching minimal levels after 3 days. Although glucose concentrations also decreased progressively under HG conditions, they remained higher throughout the experimental period. Glucose depletion appeared to accelerate as the culture progressed. Thus, despite the different initial glucose concentrations in the SG and HG groups, glucose availability changed substantially over the four-day culture period. The progressive reduction in residual glucose may reflect increased glucose consumption during extended culture, potentially associated with increased cell number and metabolic demand [21,25].

Medium pH was also monitored throughout the culture (Figure 4B). The pH gradually decreased over time under both SG and HG conditions. From day 3 onward, the medium pH was lower under HG conditions than under SG conditions. The decline in pH became more pronounced as the culture progressed. These findings show that prolonged culture was accompanied by progressive glucose depletion and a concurrent decrease in medium pH. Therefore, differences between the SG and HG groups after 4 days may reflect both the initial glucose concentrations and subsequent changes in glucose availability and the extracellular environment.

### 3.5. Effects of Daily Medium Replacement on Glucose Availability and Culture Medium Conditions

Glucose concentrations progressively decreased during the four-day culture period without medium replacement. Thus, the mitochondrial alterations observed under HG conditions may reflect the initial glucose concentration, subsequent changes in glucose availability, or both. To examine these possibilities, a separate experiment was performed in which the medium was replaced every 24 h with a fresh medium containing the corresponding glucose concentration. In this experiment, cells cultured under standard-glucose conditions with daily medium replacement were designated as ESG (standard-glucose), whereas those cultured under high-glucose conditions with daily medium replacement were designated as EHG (high glucose).

**Figure 5:**
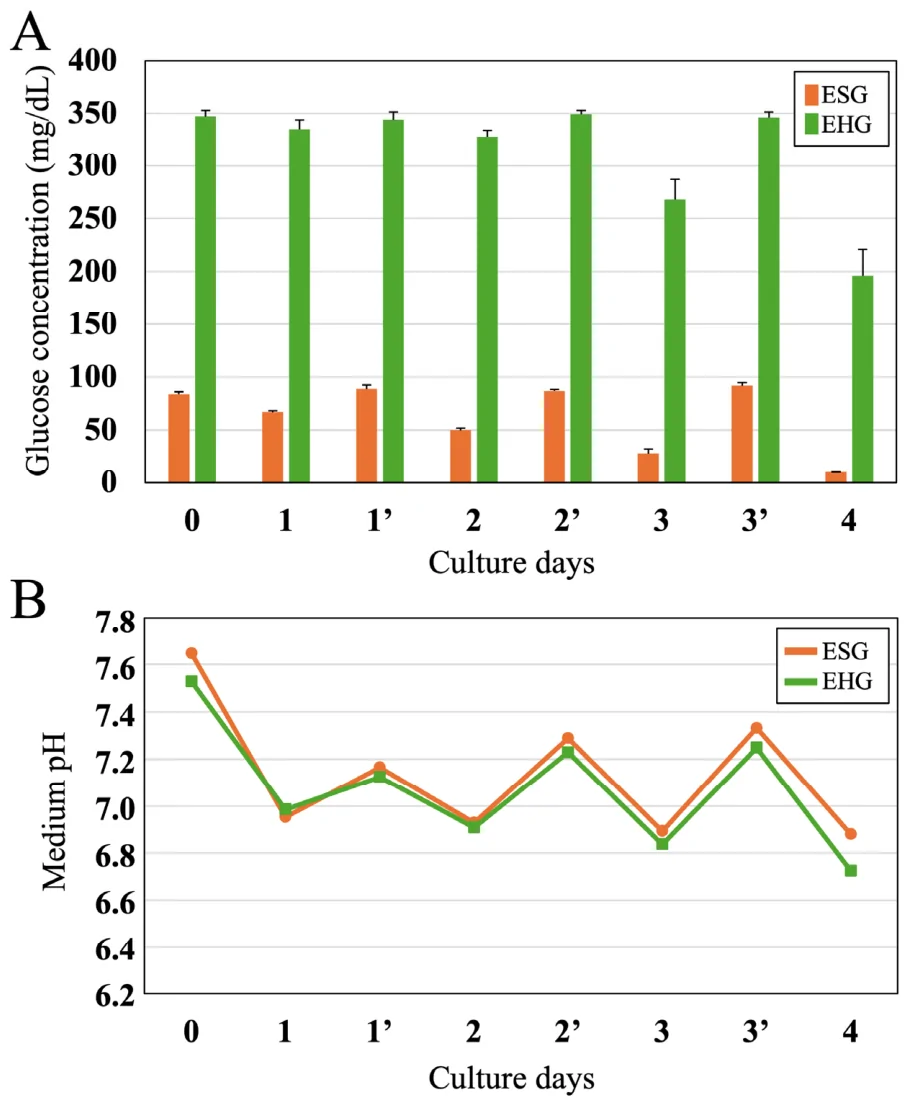
Changes in residual glucose concentration and medium pH with daily medium replacement. HEK293 cells were cultured under standard-glucose (ESG) or high-glucose (EHG) conditions, with medium replacement every 24 h. (**A**) Residual glucose concentration. (**B**) Medium pH. An apostrophe (‘) after the day number on the horizontal axis indicates a measurement obtained immediately after medium replacement; day numbers without an apostrophe indicate measurements obtained immediately before replacement. Data are presented as mean ± SD (n = 3).

Glucose concentration was monitored throughout the experiment. After each daily medium replacement, the glucose concentrations returned to their initial levels and decreased over the next 24 h (Figure 5A). Daily medium replacement repeatedly restored the designated glucose concentration during culture. Glucose concentrations decreased during each 24 h interval, and the extent of depletion appeared to increase as the culture progressed. This pattern was consistent with greater glucose consumption at later time points, although the cell number was not measured.

Medium pH was monitored concurrently (Figure 5B). After each daily medium replacement, pH increased and then decreased over the following 24 h. With daily medium replacement, medium pH did not differ significantly between the ESG and EHG groups. The pH was generally lower immediately before and immediately after medium replacement, and the decline appeared to accelerate as culture progressed.

These findings show that daily medium replacement repeatedly restored the specified glucose concentrations, allowing glucose depletion to be evaluated over successive 24 h intervals. The results also showed that the extracellular environment continued to change within each interval, with glucose depletion and reductions in medium pH becoming more pronounced over time.

### 3.6. Effects of Daily Medium Replacement on Mitochondrial Protein Abundance

**Figure 6:**
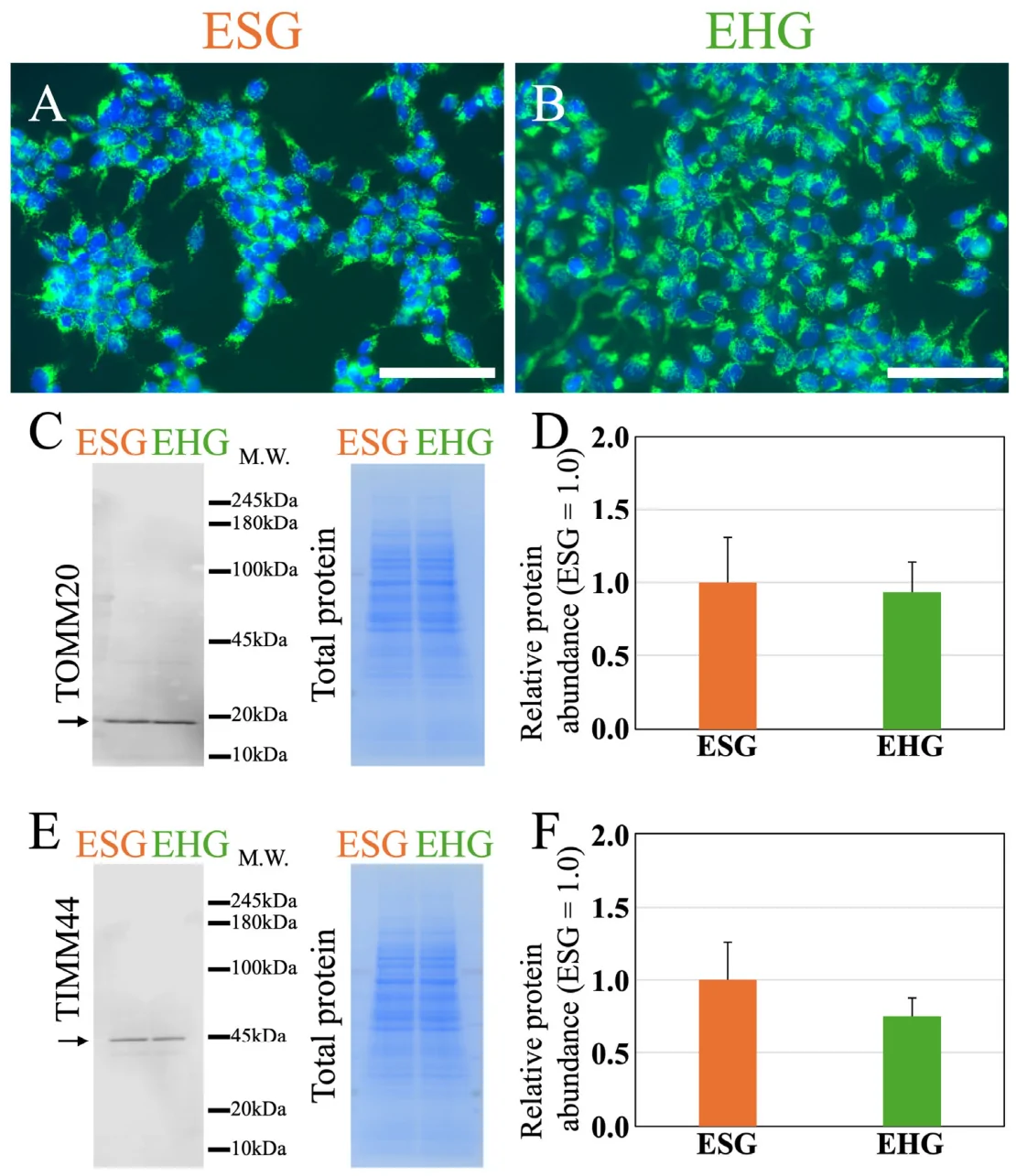
Effects of daily medium replacement on TOMM20 and TIMM44 protein abundance in HEK293 cells. Cells were cultured under standard-glucose (ESG) or high-glucose (EHG) conditions for 4 days, with the medium replaced every 24 h. (**A**,**B**) Representative TOMM20 immunofluorescence images. The nuclei were counterstained with DAPI. (**C**) TOMM20 Western blot and corresponding total protein staining with EzStain AQua MEM. The arrow indicates the TOMM20 band at approximately 20 kDa used for densitometric quantification. (**D**) TOMM20 abundance normalized to the EzStain AQua MEM total-protein signal and expressed relative to the ESG group, which was set to 1.0. (**E**) TIMM44 Western blot and corresponding total protein staining with EzStain AQua MEM. The arrow indicates the TIMM44 band at approximately 44 kDa used for densitometric quantification. (**F**) TIMM44 abundance was normalized to the total protein signal and expressed relative to the ESG group, which was set to 1.0. EzStain AQua MEM total protein staining served as the loading control, and the target protein band intensities were normalized to the total protein signal. Representative immunofluorescence images are shown for qualitative assessment; fluorescence intensity was not quantitatively analyzed. M.W. indicates molecular weight in kilodaltons (kDa). Data are presented as mean ± SD (n = 4 independent samples per group). TOMM20, *p* = 0.772; TIMM44, *p* = 0.181. Scale bars, 50 μm.

TOMM20 immunofluorescence was examined after 4 days of culture with daily medium replacement. Unlike the more intense TOMM20 fluorescence observed in HG cells cultured without medium replacement, TOMM20 fluorescence did not visibly differ between the ESG and EHG groups when the medium was replaced every 24 h (Figure 6A,B). Western blotting produced consistent results (Figure 6C). With daily medium replacement, total protein-normalized TOMM20 abundance did not differ significantly between the ESG and EHG groups (Figure 6D; n = 4; *p* = 0.772). TIMM44 abundance was also evaluated by Western blotting. Similarly, total protein-normalized TIMM44 abundance did not differ significantly between the ESG and EHG groups (Figure 6E,F; n = 4; *p* = 0.181).

Overall, no statistically significant difference in TOMM20 abundance was detected between the ESG and EHG groups after daily medium replacement. TIMM44 abundance did not differ significantly between the groups under these conditions.

### 3.7. Effects of Daily Medium Replacement on Mitochondrial Morphology

**Figure 7:**
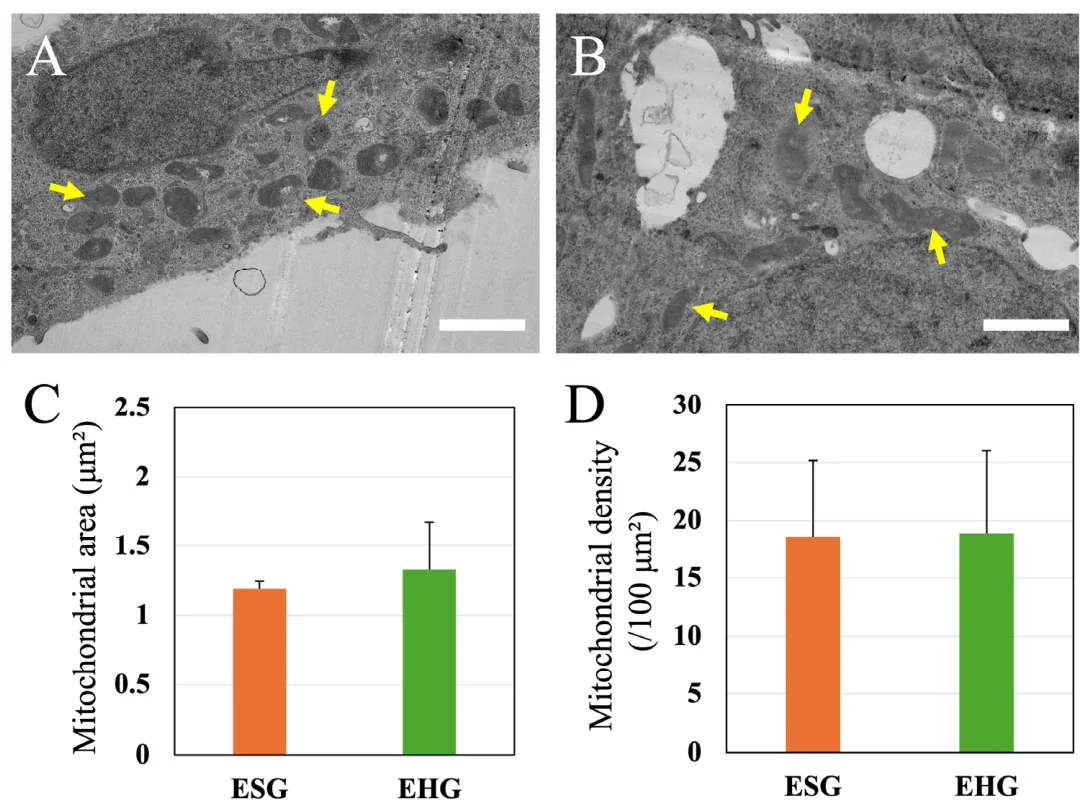
Effects of daily medium replacement on mitochondrial morphology and density in HEK293 cells. (**A**,**B**) Representative transmission electron microscopy (TEM) images of HEK293 cells cultured under standard-glucose with daily medium replacement (ESG) or high-glucose with daily medium replacement (EHG) conditions for 4 days. Arrows indicate mitochondria. (**C**) Quantification of the mitochondrial cross-sectional area. (**D**) Quantification of mitochondrial density, expressed as the number of mitochondria per 100 μm^2^ of the cellular area. Data are presented as mean ± SD (n = 3 independent dishes per group). Scale bars, 2 μm.

Mitochondrial morphology was examined after 4 days of culture with daily medium replacement (Figure 7A,B). Mitochondrial ultrastructure was evaluated using transmission electron microscopy, and mitochondrial cross-sectional area was measured on day 4. With daily medium replacement, the mean mitochondrial area did not differ significantly between the ESG and EHG groups (Figure 7C; n = 3 independent dishes per group; *p* > 0.05). 

Mitochondrial density was quantified to determine whether the difference in mitochondrial size was accompanied by a change in the number of mitochondria per unit of cellular area (Figure 7D). Mitochondrial density did not differ significantly between the ESG and EHG groups (n = 3 independent dishes per group; *p* > 0.05). Thus, even with daily medium replacement, the difference in mitochondrial size was not accompanied by a detectable difference in mitochondrial density.

### 3.8. Comparison of Mitochondrial Morphology Across All Culture Conditions

**Figure 8:**
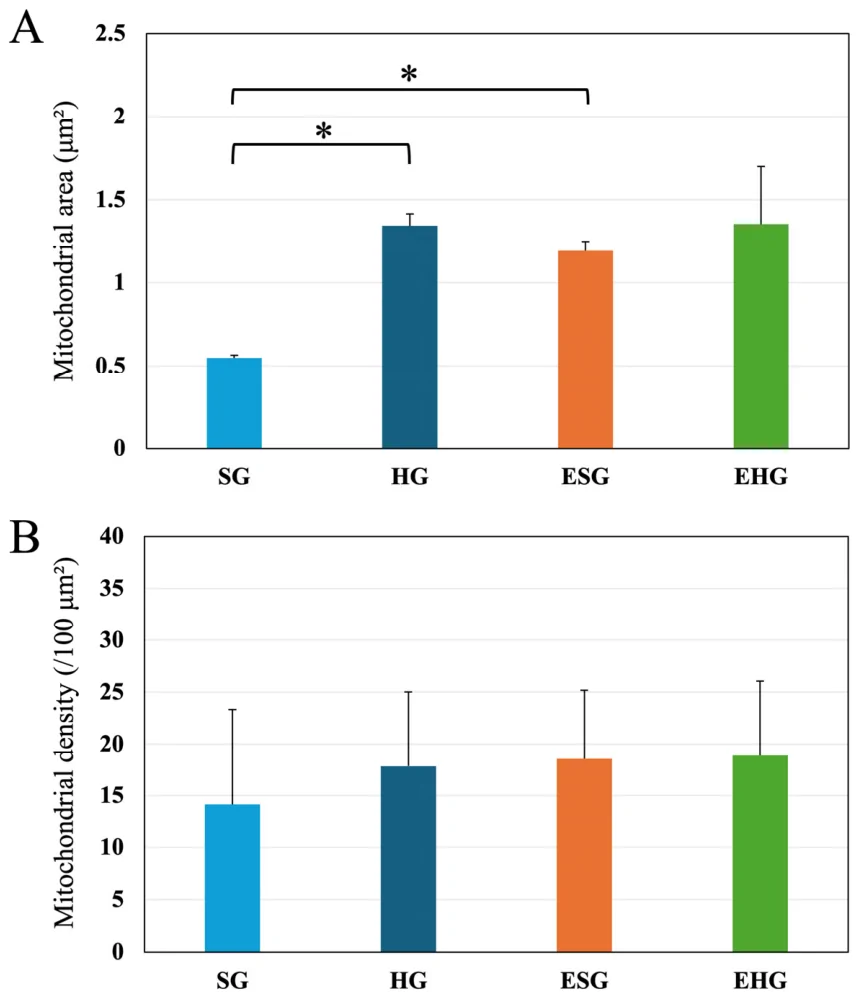
Mitochondrial cross-sectional area and mitochondrial density under different glucose and medium-replacement conditions. (**A**) Mitochondrial cross-sectional area. Data are presented as the mean ± standard deviation (SD) from three independent culture dishes per condition (n = 3). Differences among the four culture conditions were analyzed using one-way ANOVA followed by Tukey’s multiple-comparison test. The effects of glucose concentration and medium-replacement condition, as well as their interaction, were additionally evaluated using two-way ANOVA. (**B**) Mitochondrial density. Individual cells were measured within each dish, and the mean value for each dish was used as the independent observation (n = 3 dishes per condition). A *p*-value < 0.05 was considered statistically significant. Asterisks indicate statistically significant differences between the groups indicated in the figure.

To further compare mitochondrial morphology across different culture conditions, mitochondrial cross-sectional area and density were evaluated in the four experimental groups: SG, HG, ESG, and EHG (Figure 8). Mitochondrial cross-sectional area was significantly greater in the HG group than in the SG group under conditions without medium replacement (*p* < 0.05). With daily medium replacement, mitochondrial cross-sectional area did not differ significantly between the EHG and ESG groups.

To evaluate the effects of glucose concentration and medium-replacement condition on mitochondrial cross-sectional area, two-way ANOVA was performed (Figure 8A). Significant main effects were observed for glucose concentration (F(1, 8) = 21.595, *p* = 0.0017) and medium-replacement condition (F(1, 8) = 10.067, *p* = 0.0131). A significant interaction between glucose concentration and medium-replacement condition was also observed (F(1, 8) = 9.461, *p* = 0.0152), indicating that the effect of glucose concentration on mitochondrial cross-sectional area differed depending on the medium-replacement condition. Although mitochondrial cross-sectional area was significantly greater under HG conditions than under SG conditions both with and without daily medium replacement, the difference between the glucose conditions was smaller with daily medium replacement.

In contrast, mitochondrial density, expressed as the number of mitochondria per 100 μm^2^ of cellular area, did not differ significantly between the SG and HG groups without medium replacement or between the ESG and EHG groups with daily medium replacement (Figure 8B). Thus, the morphological differences observed among the experimental conditions were characterized primarily by changes in mitochondrial cross-sectional area, without detectable differences in mitochondrial density between the glucose conditions within either medium-replacement condition.

## 4. Discussion

We examined cellular morphology and mitochondrial characteristics in HEK293 cells cultured for 4 days under different glucose conditions. Without medium replacement, prolonged culture under high-glucose (HG) conditions was associated with greater TOMM20 and TIMM44 abundance and a larger mean mitochondrial cross-sectional area than culture under standard-glucose (SG) conditions. During this period, however, medium glucose concentrations progressively decreased and pH changed. When the medium was replaced every 24 h, no statistically significant differences in TOMM20 or TIMM44 abundance were detected between the SG and HG groups. The between-group difference in mitochondrial cross-sectional area was also smaller than that observed without medium replacement. These results suggest that mitochondrial responses during prolonged HG culture are associated with both the initial glucose concentration and subsequent changes in glucose availability and the extracellular environment. As cell number and cellular biomass were not measured during the culture period, changes in residual extracellular glucose concentration could not be normalized to cell number or biomass. Therefore, the present data do not establish glucose consumption per cell or distinguish differences in glucose utilization from possible differences in cell proliferation or survival. An important limitation of this study is that daily medium replacement affects not only glucose availability but also the concentrations of other nutrients and metabolites and the extracellular pH. Therefore, the observed differences between the medium-replacement conditions cannot be attributed solely to glucose availability.

A principal finding was the increased abundance of TOMM20 and TIMM44 in cells cultured under HG conditions for 4 days without medium replacement. TOMM20 is an outer mitochondrial membrane component of the translocase of the outer membrane (TOM) complex that recognizes and initiates the translocation of mitochondrial precursor proteins. TIMM44 is associated with the translocase of the inner membrane (TIM23) machinery and contributes to protein import across the inner mitochondrial membrane. The concurrent increases in TOMM20 and TIMM44 abundance suggest that prolonged HG culture may alter mitochondrial protein homeostasis and protein import machinery [12,13,14]. However, increased TOMM20 and TIMM44 abundance does not directly demonstrate an increase in mitochondrial number. Mitochondrial protein abundance may reflect mitochondrial number, the concentrations of individual mitochondrial proteins, or changes in mitochondrial state [16,17,18]. These findings support the interpretation that mitochondrial protein abundance and mitochondrial number or density are not necessarily equivalent measures of mitochondrial content.

The increase in mitochondrial protein abundance was accompanied by a significantly greater mean mitochondrial cross-sectional area under HG conditions. In contrast, mitochondrial density did not differ significantly between the SG and HG groups. Together, these findings suggest that the morphological changes associated with prolonged HG culture were characterized primarily by the enlargement of individual mitochondria rather than an increase in the number of mitochondria per unit cellular area. Because dish-level means were used for statistical analysis, the independent experimental units were culture dishes rather than individual mitochondria or cells. Mitochondrial enlargement under high-glucose conditions has been reported in other cellular models [6,7,8]. The mitochondrial enlargement observed under HG conditions suggests that prolonged exposure to this culture environment is associated with structural remodeling [26,27]. Mitochondrial cross-sectional area measured via TEM is influenced by the orientation of the mitochondria within the section and, therefore, does not directly represent mitochondrial volume or total mitochondrial mass. No statistically significant difference in mitochondrial density was detected between the experimental conditions. However, this finding does not establish that mitochondrial number remained unchanged or that mitochondria were lost under the low-glucose condition. As mitochondrial density was assessed in two-dimensional images, the measurements may also be influenced by mitochondrial morphology, distribution, and the plane of section. Further analyses would be required to determine whether mitochondrial turnover or changes in mitochondrial number contributed to the observed findings.

Mitochondrial distribution and dynamics were not examined via live-cell fluorescence imaging or flow cytometry. Although these approaches could provide complementary information about mitochondrial morphology and distribution, fluorescence-based measurements may be influenced by probe characteristics and mitochondrial membrane potential. Therefore, additional imaging approaches, together with appropriate validation, will be useful for further characterizing mitochondrial changes during prolonged culture under different glucose conditions. Nevertheless, mitochondrial enlargement does not necessarily indicate improved mitochondrial function or “mitochondrial activation” [5,10,26]. Mitochondrial respiration, oxygen consumption rate (OCR), ATP production, membrane potential, and reactive oxygen species (ROS) generation were not directly evaluated. Therefore, whether the enlargement represents an adaptive or compensatory response to metabolic stress remains unclear. Studies integrating morphological and functional measurements are needed to clarify the physiological implications of this remodeling.

Monitoring medium glucose concentrations throughout the culture period was central to interpreting the mitochondrial findings. Without medium replacement, residual glucose concentrations progressively decreased in both the SG and HG cultures. The decline was more pronounced in the SG group, whereas the glucose concentrations remained higher in the HG group. Thus, prolonged culture conditions cannot be characterized solely by initial glucose concentrations of 5.5 mM for SG and 25 mM for HG. The extracellular glucose environment changed substantially over a four-day culture period. Consequently, the differences in mitochondrial protein abundance and morphology between the SG and HG cultures without medium replacement may reflect both the initial glucose concentrations and subsequent changes in glucose availability [19,20,23]. This distinction is important when interpreting prolonged in vitro glucose exposure experiments because cellular nutrient consumption progressively alters the extracellular environment. As the osmolality of the culture media was not directly measured and an osmolality-matched control was not included, a contribution of differences in osmolality to the observed cellular changes cannot be completely excluded. Therefore, the observed differences should be interpreted as changes associated with the glucose culture conditions rather than as effects attributable exclusively to glucose metabolism.

The medium pH also changed during prolonged culture. The medium pH progressively decreased, with a more pronounced decline later in culture. These pH changes may reflect altered cellular metabolism and accumulation of metabolic byproducts during prolonged culture [21,28]. However, because metabolites such as lactate were not measured, the mechanisms underlying the pH changes remain undetermined. Therefore, the pH changes should be interpreted as indicators of an altered extracellular environment rather than direct evidence of increased glycolytic activity. Together, these findings show that prolonged culture under different glucose conditions was accompanied by extracellular changes beyond glucose concentration alone.

Daily medium replacement was used to examine whether the mitochondrial alterations observed after 4 days were associated with the initial glucose concentration or with changes in the culture environment over time. With daily medium replacement, no statistically significant differences in TOMM20 or TIMM44 abundance were observed between the ESG and EHG groups. Consistent with the Western blot results, TOMM20 immunofluorescence showed no visible differences between the groups. These findings suggest that the increases in TOMM20 and TIMM44 observed under HG conditions without medium replacement cannot be attributed solely to the initial glucose concentration. Instead, changes in mitochondrial protein abundance may also be associated with the evolving extracellular environment during prolonged culture. Without medium replacement, glucose is progressively depleted, while metabolic byproducts and other extracellular factors may accumulate, and medium pH may change. Daily medium replacement replenishes glucose and removes accumulated extracellular components, thereby reducing their cumulative effects [25,28]. Daily medium replacement replenished glucose while simultaneously replenishing nutrients and removing accumulated metabolic byproducts and other extracellular factors. Therefore, the observed differences between cultures with and without daily medium replacement cannot be attributed exclusively to changes in glucose availability. In addition, because lactate concentrations were not measured and glucose availability and medium pH were not independently controlled, the individual contributions of these factors remain unresolved.

This experiment could not distinguish the contribution of glucose depletion from that of metabolite accumulation and other changes in the extracellular environment [19,20,23,28]. Medium replacement simultaneously replenished glucose and removed accumulated metabolites and soluble factors. Therefore, the increases in TOMM20 and TIMM44 abundance cannot be attributed specifically to glucose depletion. Rather, the findings suggest that the overall extracellular environment—including glucose availability and other medium-derived factors—may contribute to mitochondrial remodeling during prolonged culture. In this study, TOMM20 and TIMM44 were evaluated at the protein level by Western blotting, whereas their mRNA expression levels were not measured. Therefore, it remains unclear whether the observed changes in protein abundance were accompanied by corresponding changes in transcript levels. Future studies incorporating mRNA expression analysis will be required to distinguish transcriptional regulation from changes occurring at the post-transcriptional or protein level.

Changes in mitochondrial morphology did not fully parallel changes in mitochondrial protein abundance. With daily medium replacement, mitochondrial cross-sectional area did not differ significantly between the ESG and EHG groups, and values in both groups were comparable to those in the HG group without medium replacement. Mitochondrial density did not differ significantly between the ESG and EHG groups. Thus, mitochondrial enlargement persisted under daily medium replacement, without a detectable change in the number of mitochondria per unit cellular area. These findings further indicate that mitochondrial morphology can change independently of detectable changes in mitochondrial density or TOMM20/TIMM44 abundance. This partial dissociation suggests that mitochondrial morphology and protein abundance may not be regulated identically in response to the extracellular nutrient environment [2,29]. The persistent morphological difference, despite no detectable difference in TOMM20 or TIMM44 abundance, may reflect both cumulative changes during prolonged culture and glucose availability within each culture interval. Therefore, the findings suggest that mitochondrial structural remodeling and changes in mitochondrial protein abundance are related but not necessarily identical responses to the glucose environment.

Collectively, our findings support the interpretation that mitochondrial responses during prolonged HG culture are associated with both the initial glucose concentration and dynamic changes in the extracellular environment. Without medium replacement, HG culture was associated with increased TOMM20 and TIMM44 abundance and mitochondrial enlargement. Concurrently, glucose concentrations progressively decreased, and the extracellular environment changed over 4 days. However, with daily medium replacement, no statistically significant differences in TOMM20 or TIMM44 abundance were detected between the ESG and EHG groups.

The findings suggest that mitochondrial characteristics are associated with not only the initial extracellular glucose concentration but also the dynamic environment encountered during culture. These responses may involve changes in mitochondrial protein composition and morphology, which reflect mitochondrial remodeling. However, the present data do not establish whether these changes represent metabolic adaptation or a compensatory response to metabolic stress. Further functional studies are needed to determine the biological implications of the observed mitochondrial remodeling.

## 5. Study Limitations and Future Directions

This study has some limitations. First, the experiments used HEK293 cells, an immortalized cell line derived from human embryonic kidney tissue. Although HEK293 cells are widely used in cellular and metabolic research because of their robust growth, their metabolic characteristics may not represent those of differentiated cells or other transformed and malignant cell types. The generalizability of the observed mitochondrial remodeling to other cell types and species remains uncertain. Cell number, proliferation, and viability were not quantitatively assessed during the culture period. Consequently, we could not exclude the possibility that differences in cell growth or survival contributed to the changes in residual extracellular glucose concentration, medium pH, cellular morphology, and TOMM20/TIMM44 protein abundance. In addition, glucose depletion was not normalized to cell number or cellular biomass. These factors should be considered when interpreting the observed differences among culture conditions. Live-cell imaging was not performed in this study. Therefore, the temporal progression of morphological changes could not be tracked in individual cells, and the sequence of events leading to the observed morphological alterations remains unresolved. Future studies using live-cell imaging and phase-contrast microscopy may help characterize these changes more precisely. The present study examined prolonged culture in low-glucose DMEM containing serum and did not include glucose-free or serum-free conditions. These conditions may induce cellular responses that differ from those observed during culture in low-glucose medium. As apoptosis and other cell-death pathways were not directly assessed, the contribution of cell death to the observed morphological and protein-expression changes remains unresolved.

Second, although mitochondrial density was quantified as the number of mitochondria per unit of cellular area, this measurement does not represent the absolute number or total mitochondrial mass per cell. Therefore, the present analysis provides evidence that the number of mitochondria per unit cellular area did not differ significantly among the examined conditions; however, it does not exclude changes in the total mitochondrial content associated with changes in cell size or mitochondrial volume. In addition, the analysis was based on two-dimensional TEM sections and therefore did not directly measure the three-dimensional mitochondrial network. Complementary approaches are required to determine how changes in mitochondrial size and protein abundance relate to total mitochondrial content and three-dimensional mitochondrial organization.

Third, although transmission electron microscopy revealed mitochondrial enlargement, its functional implications remain uncertain. Mitochondrial respiration, oxygen consumption rate, ATP production, membrane potential, reactive oxygen species (ROS) generation, and respiratory capacity were not directly evaluated [11]. Therefore, mitochondrial enlargement should not be interpreted as evidence of enhanced or impaired mitochondrial function. Combining ultrastructural analysis with functional assays may clarify the physiological significance of this morphological change.

Fourth, the daily medium-replacement experiments have an inherent interpretive limitation. This procedure replenished glucose while simultaneously removing accumulated metabolites and other extracellular factors. Consequently, the design could not distinguish the effects of glucose depletion from those of metabolite accumulation or other extracellular changes. Future studies should measure metabolic byproducts, such as lactate, under controlled glucose conditions to identify factors associated with mitochondrial responses.

After daily medium replacement, no statistically significant differences in TOMM20 or TIMM44 abundance were detected between the ESG and EHG groups. Together with the differences observed without medium replacement, these findings suggest that mitochondrial morphology and protein abundance may respond differently to changes in the culture environment.. Future studies should examine whether the persistent morphological response under replenished HG conditions is associated with mitochondrial fusion and fission or changes in mitochondrial biogenesis and turnover [2,29,30]. Mitochondrial membrane potential, respiratory activity, ATP production, and protein import activity were not directly assessed in this study. Therefore, the observed changes in mitochondrial cross-sectional area and TOMM20/TIMM44 protein abundance cannot be interpreted as direct evidence of altered mitochondrial function. Further studies incorporating functional assays are required to determine the physiological significance of these morphological and molecular changes. These analyses should also be extended to differentiated cells and cancer cell lines with distinct metabolic phenotypes. Integrating transmission electron microscopy with immunocytochemical, biochemical, and functional analyses could clarify whether the observed remodeling is generalizable across cell types or specific to particular cellular contexts [4,6,7]. Such studies may also clarify the relationships among mitochondrial ultrastructure, protein abundance, dynamics, respiratory function, and cellular energy metabolism [14,26].

Overall, the findings in HEK293 cells suggest that mitochondrial responses during prolonged HG culture are associated with a dynamic extracellular environment and the initial glucose concentration. Studies using diverse cellular models and integrated molecular, ultrastructural, and functional approaches are required to assess the generalizability and biological significance of these responses.

## 6. Conclusions

In HEK293 cells, prolonged culture under high-glucose conditions was associated with increased TOMM20 and TIMM44 abundance and mitochondrial enlargement, but not with a detectable increase in mitochondrial density. With daily medium replacement, no statistically significant differences in TOMM20 or TIMM44 abundance were detected. No difference in mitochondrial size was detected between the glucose conditions, morover mitochondrial density remained unchanged. These findings suggest that mitochondrial size and protein abundance respond to prolonged culture conditions, whereas mitochondrial density did not differ significantly between the glucose conditions. These responses were associated with both the initial glucose concentration and changes in the extracellular environment. Therefore, temporal changes in the culture environment should be considered when assessing mitochondrial responses to glucose conditions in vitro.

## Data Availability

The raw data supporting the conclusions of this article will be made available by the authors on request.
